# Comparison of Masimo Total Hemoglobin SpHb® continuous non-invasive hemoglobin monitoring device with laboratory complete blood count measurement using venous sample: Protocol for an observational substudy of the Pregnancy Risk and Infant Surveillance and Measurement Alliance Maternal and Newborn Health (PRISMA MNH) study

**DOI:** 10.12688/gatesopenres.14499.2

**Published:** 2024-02-05

**Authors:** Fouzia Farooq, Emily R. Smith, Qing Pan, Sasha Glass Baumann, Victor Akelo, Fyezah Jehan, Margaret Kasaro, Imran Nisar, Gregory Ouma, Bellington Vwalika, M. Bridget Spelke, Joan T. Price, Zahra Hoodbhoy

**Affiliations:** 1 Department of Global Health, Milken Institute School of Public Health, George Washington University, Washington, DC, 20052, USA; 2 Department of Statistics, Columbian College of Arts & Sciences, George Washington University, Washington, DC, 20052, USA; 3 Centers for Disease Controls and Prevention - Kenya, Kisumu, Kenya; 4 Aga Khan University Hospital, Karachi, Karachi, Sindh, Pakistan; 5 UNC Global Projects Zambia, Lusaka, Zambia; 6 School of Medicine, University of North Carolina, Chapel Hill, NC, 27599, USA; 7 Centre for Global Health Research (CGHR), Kenya Medical Research Institute, Kisumu, Kenya; 8 School of Medicine, University of Zambia, Lusaka, Zambia; 9 School of Medicine, The University of North Carolina at Chapel Hill, Chapel Hill, North Carolina, NC 27599, USA

**Keywords:** hemoglobin, device validation, anemia, pregnancy, postpartum, non-invasive, Masimo

## Abstract

**Background:**

The Masimo Total Hemoglobin SpHb® is a continuous and non-invasive handheld device to measure hemoglobin levels. Previous research has found that SpHb is able to accurately detect hemoglobin levels in adult patients with a similar degree of bias and standard deviation to point-of-care invasive method measurements. Generally, limited clinical evidence, lack of validation of Masimo at higher than and lower than hemoglobin threshold values, and scientific consensus supporting the use of Masimo for accurate hemoglobin testing for the diagnosis of anemia during pregnancy calls for further research.

**Methods and analysis:**

The proposed prospective cohort will be nested within the ongoing Pregnancy Risk and Infant Surveillance and Measurement Alliance (PRISMA) Maternal and Newborn Health (MNH) study. Three study sites (located in Zambia, Kenya, and Pakistan) will participate and collect hemoglobin data at five time points (<20 weeks, 20 weeks, 28 weeks, 36 weeks’ gestation, and six weeks postpartum). We will measure hemoglobin using a venous blood sample via hematology auto-analyzer complete blood count (gold standard) and the non-invasive device. The primary objective is to assess agreement between Masimo total hemoglobin and complete blood count and on a continuous scale using Intraclass Correlation Coefficient and Bland-Altman Analysis. The second objective is to assess agreement between the two measures on a binary scale using Positive Percentage Agreement and Negative Percentage Agreement, Cohen’s Kappa, and McNemar Test. On an ordinal scale, agreement will be measured using Weighted Cohen’s Kappa and Harrel’s Concordance Index. Lastly, we will assess factors that might affect the accuracy of Masimo total hemoglobin using linear mixed models.

**Conclusions:**

The primary aim of this study is to assess the validity of the non-invasive Masimo device compared to the gold standard method of invasive hemoglobin measurements during pregnancy and postpartum periods for the diagnosis of anemia.

## Introduction

### Background

The Masimo Total Hemoglobin SpHb® is a continuous and non-invasive handheld device with an optical sensor placed on the finger that measures hemoglobin levels using pulse oximetry. The measurement takes under one minute and does not require blood samples or laboratory testing. These characteristics make it a particularly promising medical technology for resource-constrained contexts. Previous research studies have found that SpHb is able to accurately detect hemoglobin levels in adult patients with a similar degree of bias and standard deviation to point-of-care (PoC) invasive method (e.g., HemoCue device) measurements, as compared to the gold standard laboratory total hemoglobin complete blood count (CBC) method as a reference ^1– 3^. However, other evidence suggests that SpHb test accuracy is limited. A 2015 systematic review of Masimo and HemoCue performance (n=39 studies) found that SpHb had lower precision and wider 95% limits of agreement than PoC ^4^. High variability in bias and in limits of agreements for the Masimo device was also found in a study involving pregnant patients ^5^. Masimo has been approved by the US Food and Drug Administration (FDA) for use in the general population but has not been approved for use in pregnancy ^6^. The general lack of clinical evidence and scientific consensus supporting the use of Masimo for accurate hemoglobin testing for the diagnosis of anemia during pregnancy calls for further research.

### Rationale

The Pregnancy Risk and Infant Surveillance and Measurement Alliance (PRISMA) Maternal and Newborn Health (MNH) study is a population-based, open-cohort study that seeks to evaluate pregnancy risk factors and their associations with adverse pregnancy outcomes, including stillbirth, neonatal mortality and morbidity, and maternal mortality and severe morbidity. The goals are to develop a harmonized data set to improve understanding of pregnancy risk factors, vulnerabilities, and disease burden estimates in sub-Saharan Africa and Southeast Asia. Ultimately, these data will inform development of innovative strategies to optimize pregnancy outcomes for mothers and their newborns.

Anemia, a condition classified as a moderate to severe public health problem in many countries, that affects an estimated 613 million (33%) women of reproductive age worldwide, is a secondary outcome in the PRISMA MNH study ^7^. Three current PRISMA MNH study sites additionally conduct non-invasive and continuous hemoglobin monitoring with a Masimo device (located in Kenya, Pakistan, Zambia).

Producing accurate, precise, and comparable hemoglobin measurements is of special importance in pregnancy, both for clinical value in diagnosing anemia and ensuring pregnant women receive appropriate treatment. Additionally, standard of care guidelines for diagnosis of anemia and treatment are not the same across countries even when following WHO’s recommendations ^8, 9^. WHO recommends daily oral elemental iron between 30 mg to 60 mg for all pregnant women to meet increased micronutrient requirements in pregnancy and prevent maternal anemia and iron deficiency ^10^. In Pakistan and Zambia, standard of care during pregnancy is to prescribe 30-60 mg of elemental iron daily, whereas in Kenya, it is daily dose of 60 mg. In Zambia and Pakistan, if hemoglobin levels are below 11.0 g/dL, daily iron dose is increased to twice or thrice daily, depending on the severity ^10^. In Kenya, when hemoglobin levels fall between 5.1 – 8.0 g/dL, women are treated with parenteral iron, but if detected after 36 weeks, a blood transfusion may be necessary ^11^. Treatment guidelines for severe anemia vary across these countries. In Pakistan and Zambia, severe anemia is defined as <7.0 g/dL. In Pakistan, severe anemia is treated with IV iron sucrose or IV ferric carboxymaltose administered intravenously, whereas in Zambia it is treated with packed cell or blood transfusion ^10^. In Kenya, severe anemia is defined as Hb <5.0 g/dL and is treated with packed cells during pregnancy ^11^.

In our study, we will evaluate the compatibility of hemoglobin measurements between SpHb and CBC assessed via five-part autoanalyzer throughout pregnancy and at six weeks postpartum.

### Aim and objectives

The primary aim of this study is to evaluate whether total hemoglobin measures using the Masimo SpHb continuous non-invasive monitoring device are accurate as compared to gold standard CBC laboratory tests using peripheral venous blood samples. To achieve this aim, three statistical objectives were identified:

1. To estimate the level of deviation and agreement between SpHb and CBC values longitudinally.
2. To calculate the level of agreement between SpHb and CBC values among binary (healthy versus anemic) and ordinal (mild, moderate, severe anemia versus normal) measures longitudinally.
3. To describe sociodemographic and clinical factors affecting the difference between SpHb and CBC values.

## Methods

### Study design

**Figure 1.**
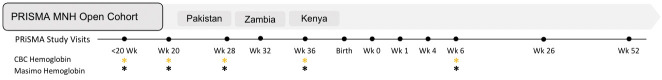
Nested prospective cohort study design.

This proposed study will use a prospective cohort design nested within the ongoing PRISMA MNH open cohort study (see Figure 1). Three sites currently using Masimo devices will participate: Lusaka, Zambia; Kisumu and Siaya, Kenya; Karachi, Pakistan. Each site has identified defined geographic regions that are appropriate for longitudinal data collection and the conduct of ongoing research involving pregnant women. Hemoglobin measurements for the validation study will occur at five timepoints: <20 weeks, 20 weeks, 28 weeks, 36 weeks’ gestation, and six weeks postpartum. The study sites in Pakistan will also collect measurements at week 32.

The PRISMA MNH study was approved by the George Washington University’s Committee on Human Research (IRB: FWA00005945) on September 30, 2022 and received local and national ethical approval in Kenya (KEMRI/CGHR/04/10/358/4166; approved September 16, 2022), Zambia (IRB00001131 of IORG0000774; approved April 8, 2022 and UNC Biomedical IRB 356795; approved August 8, 2022), and Pakistan (001-VPT-IRB-20; approved September 2, 2022). This protocol is registered with ClinicalTrials.gov (Registry number: NCT05656352, Registration date: December 19, 2022, URL of the trial in the registry database: https://clinicaltrials.gov/ct2/show/NCT05656352).

### Study sample

***Recruitment of participants***

A sample of 900 pregnant women from each site will be selected from the PRISMA study primary cohort, for a total of 2700 participants. Participants may be recruited sequentially through household-based or facility-based pregnancy surveillance (e.g., the first 900 women enrolled in PRISMA will also be included in this study from the date of protocol approval per site).

***Eligibility criteria***

Any participant that is eligible for the PRISMA study is also considered eligible for this study. However, research staff may exclude women from the study, based on the presence of injury, deformity, tattoo, or birthmark that interferes with Masimo sensor placement or performance or a finger size that does not appropriately fit the device. Presence of henna, nail polish or long nails is not an exclusion criterion, but staff will record information about these factors as they might interfere with the Masimo device performance.

***Consent***

PRISMA MNH uses a multiphase process to recruit women through pregnancy surveillance. Consent or assent will be obtained from pregnant women after a pre-screening process to determine eligibility in the PRISMA MNH study.

***Study status***

The parent PRISMA MNH study began recruiting and enrolling pregnant women on 22 September 2022 (Pakistan), 23 November 2022 (Kenya), and 15 December 2022 (Zambia). The study is planned to complete enrollment at the end of 2025. 

### Data collection

***Methods of measurement***

At enrollment, all participants selected for inclusion in the study will undergo Masimo Total Hemoglobin (SpHb) at the same time that peripheral venous blood is drawn for hemoglobin testing via CBC (gold standard method) as part of the parent study ( Table 1). At all subsequent study visits, participants will receive hemoglobin testing using both CBC and the Masimo device (SpHb). The SpHb measurement and blood draw for CBC should be done concurrently, or as close in time as is possible. Time of SpHb measurement and CBC collection and analysis will be recorded. Additionally, the IDs of the technicians collecting SpHb measurement and of technicians analyzing CBC (not the ones collecting blood specimens for CBC analysis) on the hematology analyzer will be recorded.

**Table 1.** Description of hemoglobin measurement by method.

Measurement	Biological sample	Method	Order of test
SpHb	N/A	Masimo Rad-67 ^®^ Pulse CO-Oximeter and rainbow ^®^ sensors	First (with vital signs)
CBC	Venous blood in EDTA tubes. To be analyzed within 6 hours of collection or stored at 2 to 8C and analyzed within 24 hours.	Beckman Coulter DxH520 five-part differential hematology analyzer (in Pakistan) or Sysmex XN-1000™ Hematology Analyzer five-part differential (in Zambia) or Beckman Coulter, Ac.T 5 Diff CP (Cap Pierce) (in Kenya)	Second (after SpHb)

Additional variables collected or calculated as part of the PRISMA MNH parent study that will be used in this substudy analysis can be found in Box 1, below. All data is collected by trained research staff using designated collection tools that correspond to scheduled study visits conducted either at a study facility or at the participants’ home.

**Box 1. List of maternal characteristics**

•     Maternal age,

•     Anemia severity stratum,

•     Nutritional status (e.g., iron deficiency),

•     HIV status (Zambia and Kenya sites only),

•     Malaria status (Kenya site only),

•     Helminth infection status (Kenya site only),

•     Gestational age at assessment and at first ANC visit,

•     Participant’s early pregnancy BMI (measured at first ANC visit),

•     Mid upper arm circumference (MUAC),

•     Study site,

•     Tobacco use,

•     Estimated plasma volume (PV).

**Figure 2.**
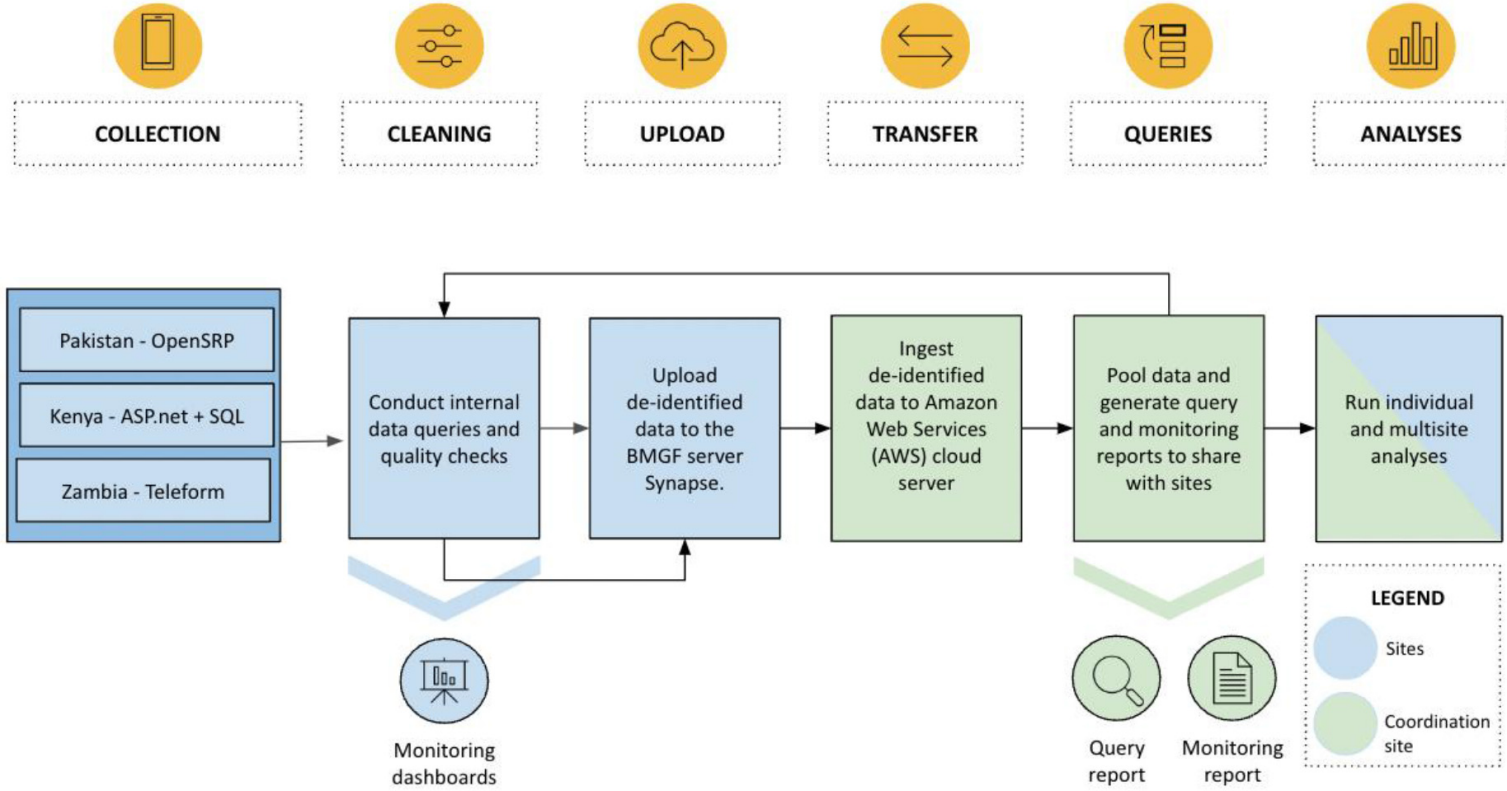
Workflow for data storage, transfer, and archiving.

Software used in the parent study are detailed in Figure 2. Three different site-specific servers (Kenya: ASP.net and SQL; Pakistan: OpenSRP; Zambia: Teleform) will be used for data collection. After the internal quality checks conducted by site data managers, de-identified data will be uploaded to the secure Bill & Melinda Gates Foundation (BMGF) server Synapse. Data will then be transferred for storage and analysis to the PRISMA Amazon Web Services (AWS) Server at the George Washington University (coordination site).

***Blinding of results***

Subject-specific results from the SpHb and CBC tests will be independently collected and technicians analyzing the SpHb will not be informed of the results of the CBC, and vice versa.

### Data quality control

***Equipment standardization and calibration***

All laboratory measurements will be conducted according to standardized operating procedures (SOPs) internally developed for the parent study. Specifically, research staff and laboratory technicians will follow exactly the Laboratory SOP, Maternal Clinical SOP, Infant Clinical SOP, and the Anthropometry Quality Assurance and Quality Control (QAQC) SOP. These SOPs describe in detail the required equipment (including test kit models) and instructions for equipment calibration and maintenance, biological specimen collection, specimen processing, sample management, and results reporting. Participating laboratories will also perform scheduled quality checks and were required to enroll in the United Kingdom National External Quality Assessment Scheme (UK NEQAS) for hematology. In addition, for the purposes of this substudy protocol, the five-part differential hemoglobin analyzer and Masimo device will be routinely calibrated according to the company's guidelines. Each Masimo device user will receive training from the company, and we will maintain a daily use log as outlined in the SOP.

***Corrective action***

SpHb: Cases with reference errors (difference between the SpHb and CBC measurements greater than 0.5 g/dL, missing paired CBC reading), user errors (incorrect device setting) and problematic signal quality checks (low signal stability, low performance improvement quality (low perfusion), low SpHb confidence (ambient light interference) - indicated as spot-check monitoring results on the instrument) will be recorded to quantify the percent of cases with failure using lab-designated SOPs.

If there are errors related to CBC, samples should be re-run in case of any errors. If errors persist, lab designated SOPs (such as a daily QC log) will be used to document the errors and appropriate personnel will be notified. Issues related to blood draw (e.g., hemolysis) will be documented for corrective actions, but more blood will not be drawn.

Statistical analysis will be performed using both per-protocol (PP) (excluding outliers and errors) (n<900) and intention-to-treat (ITT) (including outliers and errors) (n=900) analyses. Different from randomized clinical trials where PP and ITT refer to the exclusion or inclusion of data points when participants do not comply to their assigned treatments, here PP and ITT refer to the exclusion or inclusion of data points when the technician didn’t follow the protocol or user’s manual in correctly collecting blood sample or measuring hemoglobin levels, which may lead to outliers or errors.

***Missing data***

We will examine the percentage and patterns of missingness of the hemoglobin measurements. In cases where missingness is high (e.g., >10%) or cases where missing probabilities are associated with certain factors, multiple imputations (MI) will be carried out. An inclusive imputation model will be employed with all relevant risk factors and hemoglobin measurements from the same patient at different time points. MI results will be compared to the complete case analysis to test the robustness of our conclusions of missing data.

## Statistical analysis plan

All analyses will be conducted in R and STATA.

### Objective 1: Deviations/agreement between SpHb and CBC

***Intraclass correlation coefficient***

The intraclass correlation coefficient (ICC) is a measure of similarity of within-group values in clustered data ^12^. Here the ICC (with 95% CI) will be used to evaluate the degree of agreement of hemoglobin values using the degree of variances between SpHb and CBC measurements with respect to variances within SpHb measurements and variances within CBC measurements. Observed ICC with 95% CI will be reported.

***Bland–Altman analysis***

Bland–Altman (B&A) analysis is a recommended analytical method of assessing the comparability between different techniques/methods using a graphical approach using mean differences and agreement intervals. It will be used to estimate agreement between the two instruments used to measure hemoglobin levels on a continuous scale ^13, 14^. The quantification of agreement between two measurements is assessed by determining the mean difference on two quantitative measurements (i.e., the mean difference between the two observers or techniques), their standard deviation and constructing limits of agreement.

Given that multiple repeated measurements are measured on the same participant at different gestational ages, the variances in the ICC and B&A analyses will be calculated according to the data structure. For repeated measures, using the differences between SpHb and CBC measures, both, between variance and within variance will be calculated as follows: 


$$\begin{array}{c} Var(\underline{\delta }\text{.})=\frac{Var_{between}}{n}+\frac{Var_{within}}{n*n_{r}},\\ Var_{between}=\frac{\sum_{i=1}^{n}\;{\left({\underline{\delta }}_{i.}-{\underline{\delta }}_{..}\right)}^{2}}{n-1}\\ {Var}_{\mathrm{within}}=\frac{\sum_{i=1}^{n}\;\sum_{j=1}^{n_{r}}\;{\left(\delta_{ij}-{\underline{\delta }}_{i.}\right)}^{2}}{n(n_{r}-1)} \end{array}$$

Where, subscript *i* and *j* denote participant ( *i*) and repeated blood sample ( *j*), where we have *i*=1,..., *n* participants and *j*=1,..., *n _r_* repeated blood samples per participant, *δ _ij_* is the difference between the SpHb and CBC measurements from participant i blood sample j,

*δ _i_* . is the average difference of all repeated samples from participant i,

*δ* .. is the average difference from all participants and all repeated samples.

### Objective 2: Degree of agreement of SpHb and CBC

***Agreement of anemia status (binary measure)***

**Positive percentage agreement and negative percentage agreement**

Positive percentage agreement (PPA) and negative percentage agreement (NPA) measure the level of agreement between two sets of binary measures. Here, both PPA and NPA will be used to examine the level of agreement between test methods (SpHb/CBC) in identifying anemia in pregnant women (i.e. anemic/healthy). Bootstrap 95% confidence intervals will be obtained for PPA and NPA based on repeated measurements where observations from the same participant will be sampled or not sampled together.


PPA=#positive(anemia)bySpHb/#positive(anemia)byCBC

NPA=#negative(healthy)bySpHb/#negative(healthy)byCBC

**Cohen’s kappa**

Cohen’s kappa is a measure of interrater reliability, where 0 indicates agreement equivalent to chance and 1 indicates perfect agreement. For repeated measures of matched pair data, the variance of kappa can be estimated nonparametrically ^15^. In this study it will be used to assess the agreement between two procedures (CBC and SpHb) in the independent matched-pair data using defined strata. For example, if both the CBC and SpHb classify a sample as “severe deficiency”, the samples will be rated as “in agreement”. Total number of samples in agreement will be counted and Cohen’s kappa will be calculated as follows:


$$\text{k}=({\text{P}}_{\text{o}}-{\text{P}}_{\text{e}})\text{/}({\text{1-P}}_{\text{e}})$$

Where, P _o_ is the *observed* level of agreement and P _e_ is the *expected* level of agreement. Here, the observed level of agreement is the proportion of the cases the two techniques agree upon. The expected level of agreement is the proportion of agreement that is expected by change.

**McNemar test**

McNemar test is a non-parametric test used to examine the change in proportion for repeated measures of matched paired data ^16^.

The test statistic is defined such that k=1,...,K represents different time points and within the kth cluster, b _k_ and c _k_ are the number of disagreeing pairs (b _k _: anemia according to SpHb while normal according to CBC; c _k _: normal according to SpHb while anemia according to CBC). Under the null hypothesis of no difference in the two systems (SpHb, CBC) of anemia classifications, *χ* ^2^ is asymptotically distributed as a chi-square with one degree of freedom for a large number of clusters *K*→∞


$$\chi 2=\frac{K-1}{K}\frac{{\left[\sum_{k=1}^{K}\;(b_{k}-c_{k})\right]}^{2}}{\sum_{k=1}^{K}\;{\left(b_{k}-c_{k}\right)}^{2}}$$

***Agreement of anemia severity (ordinal measure)***

**Weighted Cohen’s kappa**

Weighted Cohen’s kappa is a measure of interrater reliability for ordinal or categorical items, in this case, for anemia severity (mild, moderate, severe, healthy, above normal). Linear or quadratic weights are assigned to different combinations of SpHb category (i) + CBC category (j). The equation for weighted κ is below, where i, j represents the category judging from SpHb and CBC respectively. Furthermore, w _ij_, O _ij_ and e _ij_ are elements in the weight, observed counts, and expected counts matrices, respectively. 


$$\kappa =1-\frac{\sum \;}{}\frac{\sum_{i=1}^{4}\;\;\sum_{j=1}^{4}\;w_{ij}o_{ij}}{\sum_{i=1}^{4}\;\;\sum_{j=1}^{4}\;w_{ij}e_{ij}}$$

**Harrel’s concordance index**

Harrel’s concordance I=index (C-Index) is a goodness of fit measure where the number of concordant pairs is divided by the number of comparable pairs. Comparable pairs are any pair of participants from the sample, that is, the number of comparable pairs is *n*×( *n*–1)/2. Among all comparable pairs, concordant pairs are the cases where the participant with higher rank (severe anemia=1, moderate anemia=2, mild anemia=3, healthy=4, above normal=5) using SpHb measurements is also the participant with higher rank using CBC measurements. Bootstrap confidence intervals can be similarly obtained for weighted Kappa and Harrel’s C-Index where repeated measures from the same participant will be selected or not selected together.


$$c=\frac{no.of\;concordant\;pairs}{no.of\;concordant\;pairs+no.of\;discordant\;pairs}$$

### Objective 3: Factors affecting differences between SpHb and CBC

In order to assess factors that might affect the accuracy of SpHb, we will include the following characteristics in linear mixed models in order to assess differences between paired SpHb and CBC measurements. These variables will be collected during the enrollment visit, ANC visit, and or during the postpartum visit.

- Maternal age,
- Anemia severity stratum,
- Nutritional status (e.g., iron deficiency),
- HIV status (Zambia and Kenya sites only),
- Malaria status (Kenya site only),
- Helminth infection status (Kenya site only),
- Gestational age at assessment and at first ANC visit,
- Technician,
- Time between SpHb collection and CBC collection,
- Participant’s early pregnancy BMI (measured at first ANC visit),
- Mid upper arm circumference (MUAC),
- Study site,
- Tobacco use,
- Estimated plasma volume (PV) ^17^ where Kaplan-Hakim formula ^18^ estimates PV as:

PV(cPV)=(1−hematocrit)∗[a+(b∗weightinkg)]

Where, *a = 1,530* in males and *864* in females, and

*b = 41* in males and *47.9* in females.

We will assess differences between these subgroups as follows:

*Candidate forms of the differences in hemoglobin levels (outcome):*

- Differences ( *Δ* = *SpHb*– *CBC*),
- Absolute differences | *Δ*|,
- Log(SpHb) - log(CBC) = log(SpHb/CBC).

*Comparison of the outcomes across different categories:*

- Contingency tables will be constructed where the distribution of the outcome (differences) will be compared across different levels of the risk factors. For example, mean/SD/IQR of the outcome will be listed side by side for each site and p-values using mixed models comparing the distributions between different locations will be reported.

*Plot of the outcome versus risk factors:*

- Lowess or kernel smoothing curves will be fitted for the relationship between the outcome and continuous risk factors such as true Hb values.

*Univariate analysis of candidate risk factors:*

- One linear mixed model will be fitted for each candidate risk factor. Candidate risk factors will be tested one at a time and marginal associations between the size of measurement errors and each risk factor will be reported as change per sample SD or change w.r.t. reference level, 95% confidence interval and p-values. All effect sizes and CI can be reported in a forest plot

*Multivariate analysis of selected candidate risk factors:*

- Elastic net penalized regressions (R package glmnet) with cross validation selected tuning parameters will be employed to select a set of harmonious and prognostic covariates. One multivariate linear mixed model will be fitted with control of the collinearity (VIF <8) and manual tuning using clinical knowledge. The predictive accuracy of the final model will be assessed using R ^2^ and residual plots.

### Sample size considerations

***Total sample size***

The total sample size of 900 per study site will give 90% power for site-specific analyses to detect a systematic shift (defined as mean difference between each pair of SpHb and CBC divided by the standard deviation of all paired differences) of size 0.108 between the mean of SpHb measurements and the mean of CBC measurements. That is, if the SpHb system shifts from the reference CBC measurements by 10.8% of the SD of the mean difference, the probability that we will detect such a systematic shift using one sample t-test with significance level 0.05 is 90%. The total sample size of 900 will also give 80% power to detect a systematic shift of size 0.093*SD between the mean of SpHb measurements and the mean of CBC measurements. The SD can be calculated in the same way as those in the Bland Altman plots. However, 900 is a very conservative estimate of the sample size and would be sufficient to analyze the three sites independently. Based on our pilot data from Kenya and Pakistan, the mean difference between SpHb and CBC is approximately 0.8 times its SD; even a sample size as small as 19 pairs would provide 90% power using one-sample t-test.

In detecting the extreme differences (i.e. large absolute value) between the SpHb and CBC measures on the same sample, the sample size of 900 will give 99.99% probability to detect the top 1% largest values in the distribution of all differences. It will also give 98.90% probability to detect the top 0.5% largest values in the distribution of all differences, and 93.31%, 83.50%, 59.36% probability to detect the top 0.3%, 0.2%, 0.1% largest values in the distribution of all differences, respectively. With three repeated measures from each of the 900 participants, the listed probabilities are conservative estimates of chances in catching extreme values.

***Strata sample size***

In cases where the distribution of differences between SpHb and CBC measurements differs in specific anemia strata, we speculate power and sample size for each stratum. Assuming the difference in the hemoglobin measurements of matched pairs is normally distributed with standard deviation SD, and the Type I error probability is set at 0.05, the relationship between the required sample size in the stratum and the detectable alternatives in the unit of SD is plotted above. If the true difference in the mean response of matched pairs is 0.8*SD, we will need to study 19 pairs of subjects to be able to reject the null hypothesis that this response difference is zero with probability (power) 0.90. When the mean difference is 0.5*SD, we would require a sample size of 33 pairs in the strata.

***Repeated measures***

The power of 90% can be achieved with 19 paired SpHb and CBC measurements in a cross-sectional study. We plan to collect repeated measurements from 19 participants at different time points. The longitudinal design will provide a power greater than 90%. Furthermore, longitudinal data also provide us opportunities to study the discrepancy or degree of agreement between SpHb and CBC at specific gestational age, given there are 19 matched pairs of data at any given time.

## Data Availability

No data are associated with this article.
