# Supplementary material for: Comparison of Masimo Total Hemoglobin SpHb® continuous non-invasive hemoglobin monitoring device with laboratory complete blood count measurement using venous sample: Protocol for an observational substudy of the Pregnancy Risk and Infant Surveillance and Measurement Alliance Maternal and Newborn Health (PRISMA MNH) study
Source: Gates Open Res. 2024 Feb 5;7:50. Originally published 2023 Mar 20. [Version 2] doi: 10.12688/gatesopenres.14499.2 (PMC10587393; doi:10.12688/gatesopenres.14499.2)
Supplement: Peer review history

# Reviewer report: Reviewer response for version 1

**DOI:** 10.21956/gatesopenres.15813.r35193

**Published:** 2023-10-19

**Authors:** Hanna Jonasson

**Affiliations:** 1 Linköping University, Linköping, Sweden

The study aims to evaluate the accuracy of a measuring tool for hemoglobin levels during pregnancy. The research will take place at multiple study sites and at multiple time points. Ultimately, the goal is to determine if the device can reliably diagnose anemia during pregnancy, addressing the need for further research in this area.

 The article is well written and I recommend it for indexing.

Is the study design appropriate for the research question?

Yes

Is the rationale for, and objectives of, the study clearly described?

Yes

Are sufficient details of the methods provided to allow replication by others?

Yes

Are the datasets clearly presented in a useable and accessible format?

Yes

Reviewer Expertise:

Microcirculation, public health, cardiovascular disease

I confirm that I have read this submission and believe that I have an appropriate level of expertise to confirm that it is of an acceptable scientific standard.

# Reviewer report: Reviewer response for version 1

**DOI:** 10.21956/gatesopenres.15813.r34003

**Published:** 2023-09-25

**Authors:** Armando García Guerra

**Affiliations:** 1 National Institute of Public Health, Mexico City, Mexico

General comment:

 This article is the report of a study protocol on the topic of validation of hemoglobin measurement in a study population group. This study is well planned and raises some questions that I hope will be useful in the presentation of the manuscript.

 Introduction:

 The authors note that there is a lack of clinical evidence and consensus regarding the use of Masimo during pregnancy. Although evidence is scarce, it does exist. Therefore, the suggestion in this section is to summarize what is available in this area, for example by reviewing and considering these and other articles related to this topic.

- BMC Pregnancy Childbirth. 2023 Jun 29;23(1):479. doi: 10.1186/s12884-023-05783-3.
- Anesteziol Reanimatol. 2012 Nov-Dec;(6):36-9.
- Anaesthesia. 2013 Jan;68(1):40-5. doi: 10.1111/anae.12039. Epub 2012 Oct 22.

Objective:

 The objectives of the study are clear. Although it would be worthwhile to include the study population in the general objective and analysis objective number three.

 Methods:

 The description of the methods is very detailed. A possible question is whether you considered hemoglobin concentration values to be out of range, and if so, what was the range of values you used?

 Also, do you know if you made an altitude adjustment for the hemoglobin concentration?

Is the study design appropriate for the research question?

Yes

Is the rationale for, and objectives of, the study clearly described?

Yes

Are sufficient details of the methods provided to allow replication by others?

Yes

Are the datasets clearly presented in a useable and accessible format?

Yes

Reviewer Expertise:

Nutritional deficiencies, maternal and child health, evaluation of social programs.

I confirm that I have read this submission and believe that I have an appropriate level of expertise to confirm that it is of an acceptable scientific standard.

## References

[1] : Determining the validity of non-invasive spot-check hemoglobin co-oximetry testing to detect anemia in postpartum women at a tertiary care centre, a prospective cohort study. *BMC Pregnancy Childbirth* .2023;23(1) : 10.1186/s12884-023-05783-3 479 10.1186/s12884-023-05783-3 37386388 PMC10308759

[2] : [Noninvasive total hemoglobin monitoring based on multiwave spectrophotometry in obstetrics and gynecology]. *Anesteziol Reanimatol* .2012;36-9 23662518

[3] : Evaluation of point-of-care haemoglobin measuring devices: a comparison of Radical-7™ pulse co-oximetry, HemoCue(®) and laboratory haemoglobin measurements in obstetric patients*. *Anaesthesia* .2013;68(1) : 10.1111/anae.12039 40-5 10.1111/anae.12039 23088815
